# Interplay between myeloid-derived suppressor cells (MDSCs) and Th17 cells: foe or friend?

**DOI:** 10.18632/oncotarget.8204

**Published:** 2016-03-19

**Authors:** Liang Wen, Ping Gong, Chao Liang, Dawei Shou, Baoqing Liu, Yiwen Chen, Changqian Bao, Li Chen, Xiaowei Liu, Tingbo Liang, Weihua Gong

**Affiliations:** ^1^ Department of Surgery, Second Affiliated Hospital of School of Medicine, Zhejiang University, Hangzhou City, People's Republic of China; ^2^ Department of Oncology, First Affiliated Hospital of Shihezi University School of Medicine, Shihezi City, People's Republic of China; ^3^ Division of Gastroenterology, Xiangya Second Hospital, Central South University, Hunan, People's Republic of China

**Keywords:** myeloid-derived suppressor cells, Th17 cells, tumor, autoimmunity, cytokine

## Abstract

Myeloid-derived suppressor cells (MDSCs) and Th17 cells were first discovered in the fields of cancer and autoimmunity, respectively. In recent years, their activities have been explored in other biological and pathological conditions, such as infective diseases and solid organ transplantation. However, the interplay between MDSCs and Th17 cells and the mechanism of their interaction remain obscure. This review summarized and analyzed the relationship between MDSCs and Th17 cells, both of which participate in tumor, autoimmune disease, infection and other conditions. In tumors, the increase in MDSCs at the tumor site is usually accompanied by the accumulation of Th17 cells. However, their relationship is inconsistent in different tumors. In arthritic mice or rheumatoid arthritis (RA) patients, an increase in MDSCs, which could ameliorate disease symptoms, causes decreased *IL-17A* gene expression and Th17 cells accumulation. Furthermore, we concluded that the interaction between MDSCs and Th17 cells is mainly mediated by cytokines. However, the mechanisms require further investigation. Determining the details of their interplay will provide a better understanding of immune networks and could lead to the development of immunotherapeutic strategies in the future.

## INTRODUCTION

Myeloid-derived suppressor cells (MDSCs) were discovered in cancer patients over 20 years ago [1, 2]. The suppressive activity of MDSCs on immune responses has been studied widely in recent years [3]. Derived from bone marrow, MDSCs are characterized by a heterogeneous population of immature cells. In mice, MDSCs comprise two subsets, the granulocytic or neutrophilic MDSCs (G-MDSCs, CD11b^+^Ly6G^+^Ly6C^low^) and the monocytic MDSCs (MN-MDSCs, CD11b^+^Ly6G^−^Ly6C^high^) [4–6]. In humans, their phenotypes are Lin^−^HLA^−^DR^−^CD33^+^ and CD11b^+^CD14^−^CD33^+^ [7, 8]. MDSCs can be enriched in the primary lesion, lymphoid organs and even peripheral blood among various tumor patients. MDSCs function in various conditions, such as autoimmune diseases, pathogen infections, transplant rejection and chronic inflammation [3]. Both innate and adoptive immunity can be suppressed by MDSCs. In addition, they can also exhibit non-immune biological behavior, such as promoting tumor angiogenesis [9, 10]. The expansion and activation of MDSCs are influenced by their surrounding immune factors, which originate from other activated immune cells or stromal cells [3]. For instance, IL-1β is an inducer of MDSCs expansion, and can be produced by activated MDSCs [11, 12]. MDSCs interplay with other cells through different intracellular pathways, e.g., reactive oxygen species (ROS) by G-MDSCs, arginase and NO by M-MDSCs [6, 13], and intercellular signaling factors, e.g. TGF-β [14, 15].

Th17 cells, as the third subset of T helper cells, are IL-17-producing CD4+ T cells and are abundant at mucosal interfaces [16–18]. Th17 cells have a protective effect against the clearance of extracellular bacterial and fungal infections [19]. They also participate in regulating immune responses in other pathological conditions, such as autoimmune diseases, chronic inflammation, tumors and transplant rejection [20, 21]. The activation, expansion and differentiation of Th17 cells are controlled by secretory cytokines. The synergistic effect of TGF-β plus IL-6 or IL-21 is responsible for Th17 differentiation. IL-23 is essential for Th17 cell survival and stabilization [18, 22]. Recent studies revealed that IL-23 and IL-1β could induce Th17 cells to produce IL-17 [23]. By contrast, Th17 cells can secrete IL-17A (generally regarded as IL-17), IL-17F, IL-21, IL-22, and IL-23, which allow Th17 cells to communicate with other immune or non-immune cells [22]. Given that TGF-β alone induces Foxp3^+^ T regulatory cells (Tregs) in naive T cells, TGF-β plus IL-6 could drive the differentiation of naive T cells to Th17 cells instead, indicating a reciprocal relationship between Th17 cells and Tregs [24]. However, the interplay between MDSCs and Th17s remains undefined.

MDSCs are widely recognized to suppress immune responses, while the function of Th17 cells and its secreting factor IL-17 tend to promote inflammation and immune responses. Both MDSCs and Th17 cells have been widely studied in the same pathological conditions, such as tumors and autoimmune diseases. Furthermore, some immune regulatory factors, including IL-1β and TGF-β, are simultaneously involved in the development, differentiation or activation of MDSCs and Th17 cells. Based on their biological similarities and overlapping fields, this review investigated the possible interplay between MDSCs and Th17 cells in different pathological conditions.

## MDSCS ARE ASSOCIATED WITH TH17 CELLS IN VARIOUS PATHOLOGICAL CONDITIONS

The interplay between MDSCs and Th17 cells is implicated in many pathological conditions such as tumors, autoimmune diseases, infectious diseases and transplant rejection (Table 1). It has been primarily studied in the settings of tumors and autoimmune diseases. The details can be summarized and analyzed as follows.

**Table 1:** The crosstalk between MDSCs and Th17 cells in different pathological conditions

Pathological types	Number of articles	Disease models	Publication year	References
Tumors	8	Ehrlich acites carcinoma, Colorectal cancer, Melanoma, Breast cancer, Gastrointestinal cancer, Esophageal cancer	2011-2014	[26–33, 55]
Autoimmune diseases	9	Arthritis, Encephalomyelitis, Autoimmune hepatitis, Systemic lupus erythematosus	2012-2015	[37, 39–45, 56]
Infectious diseases	2	Chagas disease (*Trypanosoma cruzi*), Cystic fibrosis lung disease (*Pseudomonas aeruginosa*)	2013-2014	[48, 49]
Transplant rejection	2	Graft-versus-host disease (GVHD), Heart transplantation	2014-2015	[50, 51]
Immune system development	1	None	2013	[52]
Only cells *in vitro*	2	None	2011, 2013	[53, 54]

## RELATIONSHIP BETWEEN MDSCS AND TH17 CELLS IN TUMORS

Research on the function of MDSCs is mainly based on tumor studies. It is widely accepted that MDSCs accumulation in the tumor micro-environment can promote tumor development by inhibiting the patient's anti-cancer immune responses [3]. Th17 cells may also be found in tumor lesions. Th17 cells are involved in both tumorigenesis and tumor eradication. However, the precise contribution of Th17 cells to tumors remains obscure [25].

In malignant carcinoma, HLA-G expression is considered as the main escape mechanism to help tumor cells circumvent immune surveillance. In melanoma, a previous study revealed the underlying mechanism of this tumor escape involved the expansion of MDSCs and the absence of Th1/Th17 cytokines [26]. The relationship between MDSCs and Th1/Th17 cells has been further explored. Research into mouse melanoma demonstrated that decreased numbers of Th17 cells could promote tumor growth, which was not associated with the accumulation or suppressive function activation of MDSCs. Instead, it was associated with a decrease of CD4+ T cells in the tumor [27]. In human colorectal cancer, accumulation and expansion of peripheral MN-MDSCs were promoted by an increase of IL-17A, which was mainly secreted by tumor-infiltrating γδT cells rather than Th17 cells [28]. The proportions of MDSCs and Th17 cells in the peripheral blood of esophageal cancer patients were both elevated. However, no obvious correlation was found [29].

By contrast, other studies revealed a close relationship between MDSCs and Th17 cells. 5-fluorouracil (5FU) is characterized by its anticancer activity, which is based on the restoration of T-cell immunity *via* the elimination of MDSCs [30]. Intriguingly, 5-FU can also drive activation of the pyrin domain containing 3 (NLRP3) inflammasome in MDSCs to promote tumor growth and cancer angiogenesis by eliciting Th17 cells and inducing IL-17 production [30]. In detail, NLRP3 dependent caspase-1 activation leads to IL-1β production, which subsequently induced IL-17 production by CD4+ T cells [30]. In Ehrlich ascites carcinoma, the increased proportion of MDSCs at the tumor site was positively correlated with accumulation of IL-17+T cells. In addition, it was revealed that MDSCs could enhance the Th17 response, which relies on cytokines secreted by MDSCs. Among these cytokines, IL-6 and TGF-β synergistically promoted IL-17 production, whereas IFN-γ inhibited IL-17 production [31]. In TGF-β receptor II knock-out mice, polyoma middle T (PyMT)-induced tumors were associated with an increased number of Th17 cells, as well as more Th17-inducing cytokines, such as TGF-β, IL-6, and IL-23. IL-17 upregulates arginase (Arg), indoleamine 2,3-dioxygenase (IDO), and cyclooxygenase-2 (COX-2) to potentiate the suppressive function of MDSCs on anti-cancer immune responses [32]. In addition, some studies focused on the relationship between IL-17 and MDSCs. In gastrointestinal cancer patients, IL-17 production correlated with circulating MDSCs levels [33]. In addition, IL-17 was required for the development and tumor-promoting activity of MDSCs in tumor bearing mice [34].

## RELATIONSHIP BETWEEN MDSCS AND TH17 CELLS IN AUTOIMMUNE DISEASES

Th17 cells were first demonstrated in organ-specific autoimmunity and are closely involved in rheumatoid arthritis (RA), multiple sclerosis and inflammatory bowel disease [22]. IL-17 levels and the number of Th17 cells are positively associated with the progress of RA, whereas MDSCs have the potential to suppress the autoimmune responses and prevent tissue injury [35]. Although the exact function of MDSCs in the expansion of Th17 cells in RA is still unclear, their interplay has been studied extensively in this disease [36]. A series of clinical and experimental studies observed that in arthritic mice or RA patients, MDSCs and Th17 cells were simultaneously expanded and could be detected from the spleen, blood, lymphoid tissues, synovial fluid and inflamed paws [37–40]. The proportion of MDSCs was positively correlated with the severity of RA and an inflammatory response of pathogenic Th17 cells. MDSCs show T cell suppressive activity and produce high levels of pro-inflammatory cytokines, including TNF-α and IL-1β. *In vitro*, both human and mouse MDSCs could promote Th17 cell polarization and differentiation in an IL-1β-dependent manner [37, 39]. Adoptive transfer of MDSCs to RA mice ameliorated the disease symptoms (delayed onset of arthritis, reduced arthritis scores, and reduced joint inflammation and damage) and caused a concomitant decrease in *IL-17A* gene expression and accumulation of Th17 cells in the spleens [39]. Furthermore, transfer of MDSCs could also significantly decrease Th17 cells in draining lymph nodes [40–42]. A clinical study also demonstrated that an increase in the number of circulating MDSCs was negatively correlated with Th17 cells in RA patients [38].

In other autoimmune conditions, it was reported that G-MDSCs were associated with experimental autoimmune encephalomyelitis (EAE) development in mice [43]. G-MDSCs in the EAE model could enhance greatly the differentiation of naive CD4+ T cell precursors into Th17 cells. They increased the numbers of Th17 cells, elevated IL-17A production and enhanced the expression of RORγt, a key transcription factor that determines Th17 differentiation. During this process, as the major source of IL-1β and TGF-β, MDSCs promote Th17 differentiation [43]. The transcription factor cyclic adenosine monophosphate-responsive element modulator α (CREMα) can trigger differentiation of T lymphocytes toward Th17 cells, thus promoting autoimmunity in systemic lupus erythematosus and lung inflammation. However, in the mouse immune-mediated hepatitis model, hepatic MDSCs that overexpressed CREMα did not induce a predominant Th17 response in intrahepatic T cells [44]. In addition, in a mouse systemic lupus erythematosus (SLE) model, blockade of IL-33 could prevent the progress of SLE, which was associated with expansion of Tregs and MDSCs, and suppression of Th17 cells [45].

## RELATIONSHIP BETWEEN MDSCS AND TH17 CELLS IN INFECTIOUS DISEASES

Th17 cells are involved in host defense against various pathogen infections [21]. However, the functions of MDSCs in infection remain obscure. It was observed that expansion of MDSCs occurs in many types of infections, implying their involvement in anti-infection immune responses [46, 47]. In an acute infection model, represented by infection with *Trypanosoma cruzi*, the causative agent of Chagas disease, MDSCs were expanded in the liver and spleen. Depletion of MDSCs resulted in a Th17 cells' response associated with very high parasitemia and mortality, although a negative relationship between MDSCs and Th1/Th17 cells was found in the spleen [48]. In a model of pseudomonas aeruginosa-induced cystic fibrosis lung disease, MDSCs were efficiently generated. They dampened T cell proliferation and the Th17 cell response, which subsequently prevented neutrophil recruitment by inhibiting IL-17 release [49].

## RELATIONSHIP BETWEEN MDSCS AND TH17 CELLS IN OTHER CONDITIONS

After bone marrow transplantation, patients with graft-*versus*-host disease (GvHD) showed an increased proportion of G-MDSCs. In addition, peripheral MN-MDSCs efficiently dampened Th1 and Th17 responses [50]. In a cardiac transplant model, our own experimental studies revealed that MDSCs, rather than Th17 cells, are closely involved in transplant tolerance using an IL-6 deficient donor [51]. In cord blood, increased G-MDSCs efficiently suppressed IFN-γ, IL-5 and IL-17 production, indicating their broad suppressive effects on Th1, Th2 and Th17 lymphocytes [52]. There are also many ex vivo experimental studies that demonstrate an indirect relationship between MDSCs and Th17 cells. Different subsets of myeloid cells isolated from human peripheral blood modulated TGF-β-dependent CD4+ T cell differentiation in different ways. Human CD14^+^HLA^−^DR^−/low^ MDSCs induced Foxp3^+^ Tregs, but not Th17 cells, which were instead induced by CD14^+^HLA^−^DR^+^ monocytes. MDSCs catalyzed the transdifferentiation of Foxp3^+^ Tregs from monocyte-induced Th17 cells [53]. In ovarian cancer cell lines, induction and stability of human Th17 cells required endogenous NOS2 and cGMP-dependent NO, which were produced by human MDSCs [54].

**Figure 1:**
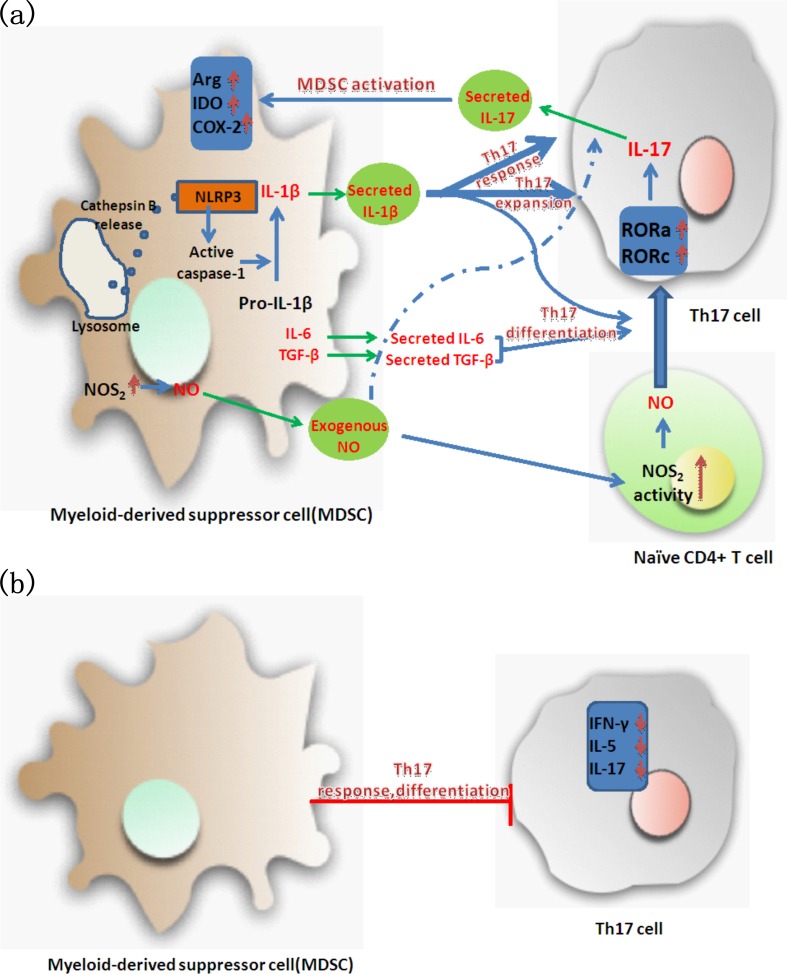
Regulation of the interaction between myeloid-derived suppressor cells (MDSCs) and Th17 cells **a.** MDSCs and Th17 cells are mutually promoted; **b.** MDSCs inhibit Th17 cells activity. Arg, arginase; IDO, indoleamine 2, 3-dioxygenase; COX-2, cyclooxygenase-2; NLRP3, pyrin domain containing-3 protein; ROR, retinoid-related orphan receptor; NOS2, nitric oxide synthase-2; NO, Nitric oxide.

Taken together, the interplay between MDSCs and Th17 cells and the underlying mechanisms of their interaction still need to be explored in depth. Interestingly, the aforementioned studies revealed that intercellular communication between MDSCs and Th17 cells was not dependent on MDSC-Th17 cell contact. Their interaction is partially mediated by secreted cytokines (Figure 1).

## CONCLUDING REMARKS AND PERSPECTIVE

In recent years, MDSCs and Th17 cells have been a focus of immunological research. They are involved in the regulation of various immune responses, including both physiological and pathological conditions, especially in the fields of tumor and autoimmunity. Recently, the association of MDSCs with Th17 cells has been studied increasingly. However, the effect of Th17 cells on MDSCs remains unclear, and its determination might increase our understanding of their interplay and other associated biological behaviors. In addition, because of the lack of studies clarifying the functions of MDSCs and Th17 cells in some non-mainstream fields, e.g., infection and transplant rejection, their potential interactions in these fields merit further investigation. To reveal the biological functions of MDSCs and Th17 cells comprehensively, further studies exploring their interactions should focus on mature disease models, such as rheumatoid arthritis. These future studies should clarify the nature of their interplay, which will increase our understanding of the immune networks and aid the development of potential immunotherapeutic strategies.
